# Deep-E Enhanced Photoacoustic Tomography Using Three-Dimensional Reconstruction for High-Quality Vascular Imaging

**DOI:** 10.3390/s22207725

**Published:** 2022-10-12

**Authors:** Wenhan Zheng, Huijuan Zhang, Chuqin Huang, Kaylin McQuillan, Huining Li, Wenyao Xu, Jun Xia

**Affiliations:** 1 Department of Biomedical Engineering, University at Buffalo North Campus, Buffalo, NY 14260, USA; 2 Department of Computer Science and Engineering, University at Buffalo North Campus, Buffalo, NY 14260, USA

**Keywords:** photoacoustic tomography, deep learning, vascular imaging, resolution improvement, 3D reconstruction

## Abstract

Linear-array-based photoacoustic computed tomography (PACT) has been widely used in vascular imaging due to its low cost and high compatibility with current ultrasound systems. However, linear-array transducers have inherent limitations for three-dimensional imaging due to the poor elevation resolution. In this study, we introduced a deep learning-assisted data process algorithm to enhance the image quality in linear-array-based PACT. Compared to our earlier study where training was performed on 2D reconstructed data, here, we utilized 2D and 3D reconstructed data to train the two networks separately. We then fused the image data from both 2D and 3D training to get features from both algorithms. The numerical and in vivo validations indicate that our approach can improve elevation resolution, recover the true size of the object, and enhance deep vessels. Our deep learning-assisted approach can be applied to translational imaging applications that require detailed visualization of vascular features.

## 1. Introduction

Over the past two decades, photoacoustic computed tomography (PACT), a hybrid imaging modality, has gained increasing interest within the field of biomedical imaging. PACT combines the rich contrast from optical absorption with the high resolution and deep imaging depth of ultrasound [1]. PACT relies on the photoacoustic effect, in which short-pulsed light beams induce local thermoelastic expansion that generates ultrasonic acoustic waves [2]. In PA, biomolecules such as melanin, lipids, and hemoglobin are used as endogenous contrasts due to their high optical absorption coefficients at specific wavelengths. Acoustic signals are detected via ultrasound transducer arrays, where they are then digitized to be used in image reconstruction [2]. Based on optical absorption of hemoglobin, PACT can visualize deep underlying vascular structures [3]. Various types of transducer arrays have been used in PACT systems. Linear-array transducers remain as one of the most popular choices due to easy integration with different light sources and compatibility with existing ultrasound systems [4]. Recently, linear transducer arrays have been used in breast imaging [5], biometrics [6], neural imaging [7], and oncology [8]. Linear arrays provide nearly isotropic resolution in the axial and lateral directions to form a B-mode image. However, these arrays have an inherently poor elevation direction resolution, which is determined by the fixed cylindrical focus of the transducer element. To maintain good sectioning capability, most linear arrays have a long elevation focus, rendering an elevation resolution that is typically a few times worse than that of their lateral and axial counterparts. This limitation directly impacts the three-dimensional performance of a PACT system [9].

Many studies have attempted to improve the elevation resolution in linear-array-based PACT through either hardware-based or software-based solutions [9]. For hardware-based methods, modifications are made to either the scanning trajectory or the detection configuration [10,11,12]. However, complicated scanning geometries lead to a longer scanning time and more data generation when compared to traditional configurations. Software-based approaches optimized the image quality through advanced image reconstruction algorithms. One example is our earlier research based on coherent weighting and focal-line 3D image reconstruction (CWFL) [13]. It computes the coherence of PA signals in 3D space and assigns the back-projected signals with a weighting factor. However, the intensity of each voxel was altered by the weighting factor, affecting the quantitative accuracy of the reconstruction. Recently, deep learning has been widely used in photoacoustic imaging studies to improve image quality and/or reduce electric noise in the raw data [14,15,16]. However, very few have attempted to improve the elevation resolution of linear-array-based PACT, due to the complexity of 3D simulation and training. Our group recently introduced Deep-E, a UNet-based method for improving the elevation resolution [17]. In that study, we converted the 3D problem into 2D space by training data only on the axial-elevation plane. Experimental validation indicated that Deep-E improved the elevation resolution by over four times. However, the input for Deep-E is still 2D reconstructed images (in the axial–lateral plane) that are stacked into 3D. As the 2D reconstruction does not consider signal divergence along the elevation direction, the quality of reconstructed images is inherently limited. 

To overcome this issue, we propose a revised Deep-E network that utilizes 3D reconstructed data as input. 3D image reconstruction is achieved by the 3D focal-line reconstruction algorithm, which considers the elevation aperture and precisely back-projects the PA signals into the 3D space [13]. As deeper signals can be seen at more scanning locations, 3D focal-line reconstruction allows better visualization of deeper structures. However, 3D focal-line reconstruction is subject to motion artifacts as it assumes that the object remains stationary during the scanning, which may last 1–2 min. Slight movements in the object might degrade the image quality. Two-dimensional reconstruction is less sensitive to motion artifacts as reconstruction is done at each imaging plane. Therefore, integrating results from both reconstruction algorithms could achieve better image quality. In the following sections, we lay out the steps to obtaining high-resolution data from both 3D and 2D reconstructions and combining them effectively. Our results indicated that, in addition to the elevation resolution improvement, the combined image contained more vascular structures and the deeper vessels were more visible.

## 2. Methods

### 2.1. System Setup

Our network was designed for the dual-scan mammoscope (DSM) photoacoustic imaging system, which was used for human breast imaging [5,18]. The transducer used in DSM was a customized 128-element linear-array transducer (IMASONIC SAS) with 2.25 MHz central frequency and >65% bandwidth. Each element in the array is arc-shaped with a 15 mm elevation length and focus at 40 mm. Light illumination is provided by a 10 Hz Nd: YAG laser (Continuum Inc., Blvd Austin, TX, USA) with 1064 nm wavelength and <10 ns pulse width. PA signals are acquired by a 256-channel data acquisition unit from Verasonics and are transmitted to the host computer for further processing. The light delivery and signal receiving are synchronized with the Q-switch output of the laser.

### 2.2. Simulation of PA Signal

**Figure 1:**
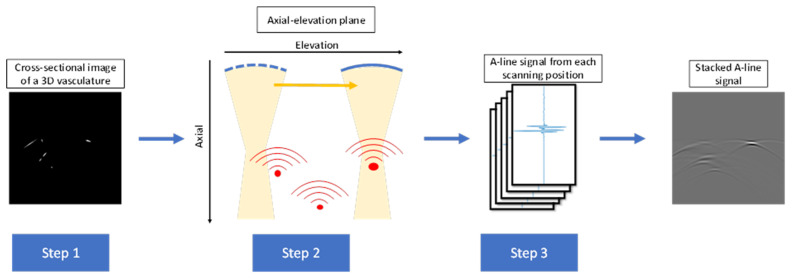
Workflow of PA simulation. Step 1: Resized PA source matrices were placed 30 mm away from the transducer element. Step 2: The transducer moved along the elevation direction to acquire acoustic signals. Step 3: Received signals at different transducer locations were stacked into one image.

We utilized the MATLAB-based photoacoustic simulation toolbox (K-wave) to generate the photoacoustic sinograms [19]. To improve the efficiency of dataset generation, we only simulated the acoustic wave propagation in the 2D space along the axial and elevation directions. This simplification is valid when the lateral resolution is much higher than the elevation resolution. We first generated the 3D vasculature matrix using the Insight Segmentation and Registration Toolkit (ITK) [20]. The toolkit generates vessels in 3D space with different diameters. We then cut the 3D vasculature matrix into 2D images. The cross-sectional images were considered as the input source for PA simulation and the ground truth for the neural network training. Figure 1 shows the workflow of 2D simulation along the axial-elevation plane. PA source matrices were resized into 50 × 50 mm with 0.1 mm pixel size and placed 30 mm away from the transducer element to mimic the experimental setup. The arc-shape transducer moved along the elevation direction at 0.1 mm step size for 500 steps to acquire acoustic signals originating from predefined PA sources. The received A-line signals at different transducer locations were stacked into one image, which preserved the primary features along the elevation direction in the raw data.

The simulated raw data were then combined with electromagnetic interference (EMI) noise acquired from the experimental system. The EMI noises are caused by electromagnetic waves emitted from external sources (e.g., laser, stepper motor) and are shown as strips along the lateral direction. Five hundred frames of EMI noise were acquired in our experimental setup and were then randomly combined with the simulated data.

### 2.3. Image Reconstruction Algorithms

The experimental data can be reconstructed using either the 2D stack or the 3D focal-line algorithms. For the 2D stack, we first reconstructed each PA B-mode image individually and then stacked them along the elevation direction based on their scanning positions. In 3D focal-line reconstruction, the data is reconstructed in 3D by considering the elevation aperture [21]. To properly back project the data in 3D, we used the focal line (a line at the linear array focus) as an auxiliary line to find out the receiving path of the photoacoustic signal. As the acoustic wavefront originating from the focal line hits the transducer surface simultaneously, for each point in 3D, we find the shortest path that goes past the focal line before reaching the transducer. The signals from each element were back-projected to a 3D space based on this principle [21].

**Figure 2:**
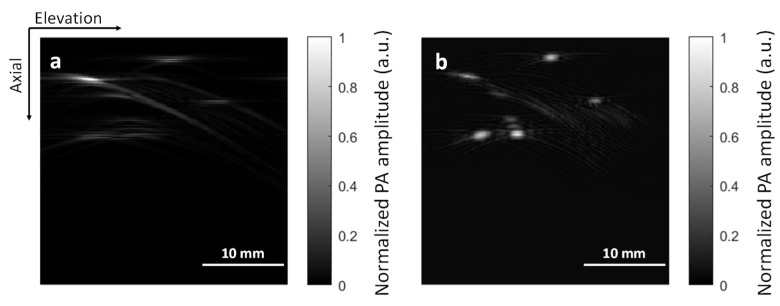
Comparison of axial-elevation plane images obtained from (**a**) the 2D stack reconstruction algorithm and (**b**) the virtual point detector reconstruction algorithm.

Image reconstruction for the simulation data is a little bit different. Because we only simulated data along the axial-elevation plane, we do not need to consider reconstruction along the lateral direction. In the Deep-E study, we simply stacked envelop-detected A-line signals along the elevation direction to mimic 2D stack-reconstructed images in the axial-elevation plane [17]. This approach cannot mimic 3D focal-line reconstruction. As we only simulate the raw data in the axial-elevation plane, the focal line turns into a focal point. Therefore, we can use the virtual point concept to reconstruct the axial-elevation raw data [22]. More specifically, for every reconstruction point in the cross-section, we found the shortest path that goes across the focal point before reaching the transducer and used this path for the time-of-flight calculation. Figure 2 compares the axial-elevation images obtained from the 2D stack and virtual-point reconstruction algorithms. It can be seen that the virtual-point reconstruction recovers features better along the elevation direction. 

### 2.4. Input Dataset and Neural Network Parameters

As mentioned above, we used the images generated by the Insight Segmentation and Registration Toolkit as the ground truth. For training, we generated six 3D vascular matrices, and each was cut into 250 images, totaling 1500. We then added four different levels of white noise to the data (signal-to-noise ratios at 6 dB, 9 dB, 12 dB, and noise-free), generating 6000 (6 × 250 × 4) images in total. We did not apply rotation to the data as we wanted to preserve the depth-dependent elevation resolution. These cross-sectional vascular data were simulated into PA raw data and reconstructed using either the 2D stack or focal-point (line) algorithms mentioned in the section above. The 2D stack data was trained using the Deep-E network mentioned in reference [17]. The focal-point data was trained using a similar fully-dense UNet (FD-UNet).

Details of the FD-UNet were mentioned in [17]. Briefly speaking, the FD-UNet is based on a CNN-based residual architecture by adding a skip connection between the input and the output [23]. First, the spatial dimensions of the feature maps are repeatedly decreased to learn local and global features related to artifact removal. Then, the learned feature maps are spatially upsampled and combined to generate an output image of the same size as the input image. 

Specifically, the input image size is 256 × 256. It first passes through a 3 × 3 convolution layer to obtain 16 feature maps. Then, it undergoes the following blocks:

- Dense block: It consists of a sequence of a 1 × 1 and 3 × 3 convolution with batch normalization and ReLU activation function. The outputs from earlier convolutional layers are concatenated together as the input to the subsequent layer.
- Down block: It is a learned downsampling operation that consists of a 1 × 1 convolution block with a stride of 1, and a 3 × 3 convolution block with a stride of 2. It gradually reduces the feature map size and increases the channel number. In the last layer of the downsampling path, we can obtain 512 feature maps with the size of 8 × 8.
- Up block: It consists of a 3 × 3 transposed convolution block with a stride of 2 followed by ReLU activation function and batch normalization to expand the feature map size.

Finally, the feature map goes through a 1 × 1 convolution layer to obtain a residual image with a size of 256 × 256. By adding the residual image with the input image, we obtain the targeted artifact-free image. To differentiate the FD-UNet used in 2D stack data training and focal-point data training, we named the first as 2D Deep-E and the latter as 3D Deep-E.

### 2.5. Image Co-Registration

After training, both the 2D Deep-E and 3D Deep-E networks can be used to improve the experimental data. However, due to the different time-of-flight calculation between 3D focal-line and 2D stack reconstruction, the two enhanced matrices cannot be perfectly overlaid and need to be co-registered to eliminate the mismatch. A MATLAB built-in function, imregdemons, is utilized to estimate the 3D displacement field and align the 2D Deep-E matrix with respect to the 3D Deep-E matrix [24,25]. After that, the aligned 2D Deep-E matrix and 3D Deep-E matrix are sliced and fused (using MATLAB function imfuse) along the axial-elevation plane to preserve features from both outputs. The fused 2D images are then stacked along the lateral direction to form the final 3D output.

### 2.6. Summary

**Figure 3:**
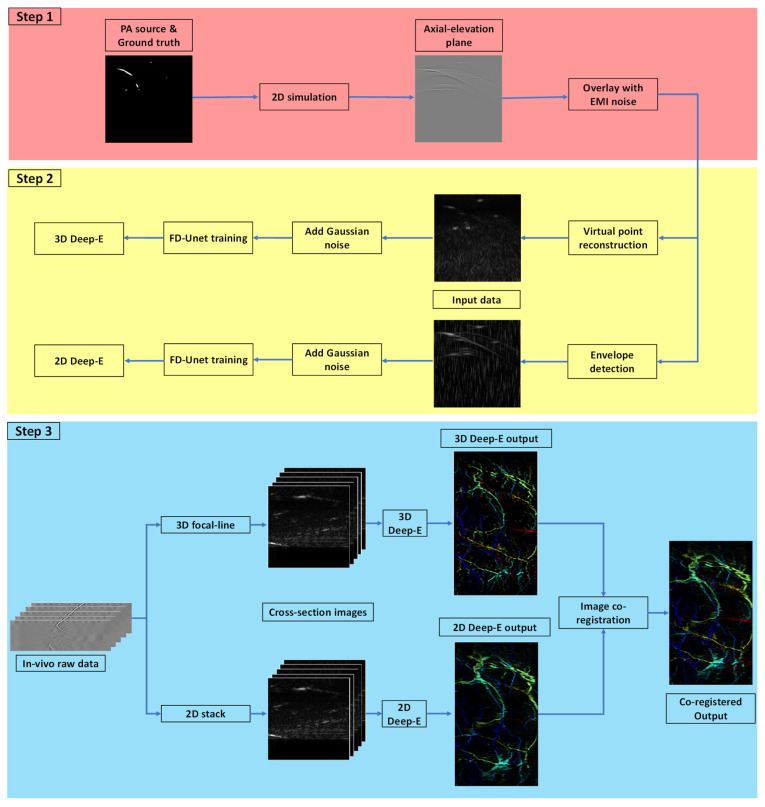
Workflow of training data generation, experimental data reconstruction, and post-training data processing.

In summary, we first simulated raw photoacoustic data in 2D space along the axial and elevation directions and combined them with the EMI noise (Step 1 in Figure 3). Second, we used envelop detection and virtual point reconstruction algorithms to reconstruct the raw data for 2D Deep-E and 3D Deep-E training, respectively. Subsequently, different levels of Gaussian noise were applied to the reconstructed data to increase the training size. We then input the images to the FD-UNet and trained the two networks separately (Step 2 in Figure 3). The total training time for each network is approximately 2 h in a workstation with AMD Ryzen 9 3950X CPU and NVIDIA GeForce RTX 2080 Ti GPU. After obtaining two trained networks, we used 2D stack and 3D focal-line algorithms to reconstruct the in vivo experimental data. The two reconstructed matrices were cut into 2D images and fed into the trained 2D deep-E and 3D deep-E networks, respectively. The output matrices from both networks were co-registered to combine the features from each algorithm (Step 3 in Figure 3).

## 3. Results

We evaluated our approach using simulation and human imaging results. First, we used simulated data with different noise levels to validate the networks’ performance in noise removal, object size recovery, and elevation resolution improvement. Then, we used the in vivo experimental data acquired from our DSM system to validate the improved imaging depth and elevation resolution.

### 3.1. Validation with Simulated Vasculature

**Figure 4:**
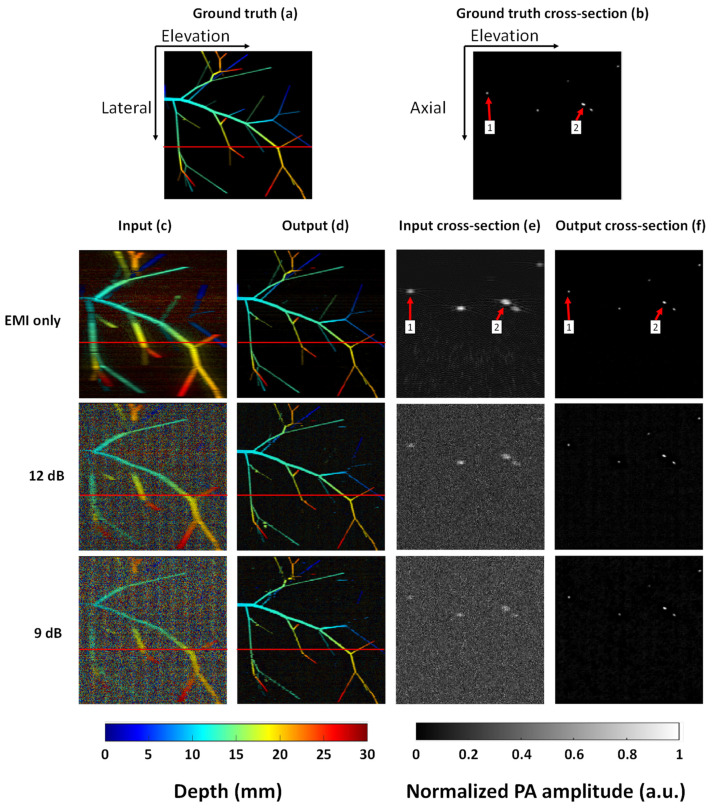
Validation through simulated data. (**a**) Ground truth of generated vasculature. (**b**) Cross-sectional image at the red line marked in (**a**). (**c**) Input images with different noise levels: EMI only, 12 dB Gaussian noise, and 9dB Gaussian noise. (**d**) Output images from the trained network. (**e**) Cross-sectional input images at the red line marked in (**c**). (**f**) Cross-sectional output images at the red line marked in (**d**).

As mentioned above, the proposed algorithm aims to improve the elevation resolution and reduce noise in the image. These can be validated through simulation data, where the ground truth is available. Figure 4a shows the max amplitude projected (MAP) ground truth, where the image is color encoded by depth ranging from blue to red (representing shallow to deep). The red lines shown in Figure 4a indicate the locations of the cross-section images in Figure 4b. Figure 4c shows the input at different white noise levels. Here, the noise level was quantified based on the Gaussian white noise only. 

Output from the network is shown in Figure 4d. It can be seen that most vessels can be clearly recovered when the SNR ratio is 12 dB or higher. When the SNR is reduced to 9 dB, some vessels with small diameters cannot be extracted. However, in most of our experimental data, the SNR is much higher than 9 dB. Figure 4e,f represent the cross-sectional images extracted from the red line in Figure 4c,d, respectively. The features shown in Figure 4e exhibited poor axial and elevation resolution. By contrast, the images shown in Figure 4f possessed high resolution in both directions. Table 1 denotes the comparison of the peak signal-to-noise ratio (PSNR) and structural similarity (SSIM) index of input and output at different Gaussian noise levels. Our proposed method improves the PSNR in all cases. The SSIM is also significantly improved, indicating high similarity to the ground truth. To validate whether our method can recover the true size of the object, we compared the axial and elevation lengths of objects 1 and 2 in Figure 4b. Table 2 shows the comparison results. The object sizes quantified from the output images are very close to the ground truth. 

### 3.2. Validation with Phantom Data

**Figure 5:**
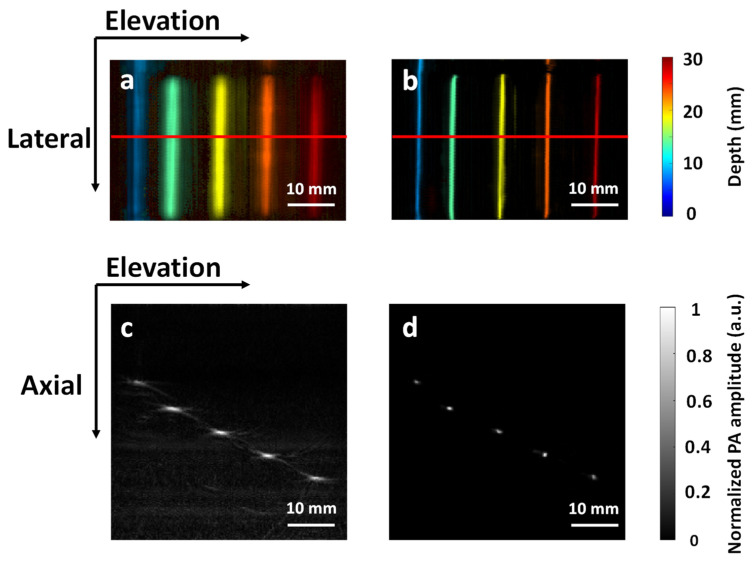
Imaging results of pencil leads at different depths. (**a**) Input reconstructed by the 3D focal-line algorithm. (**b**) Combined (2D Deep-E and 3D Deep-E) output. (**c**) Cross-sectional image obtained from the location indicated by the red line in (**a**). (**d**) Cross-sectional image obtained from the location indicated by the red line in (**d**).

**Figure 6:**
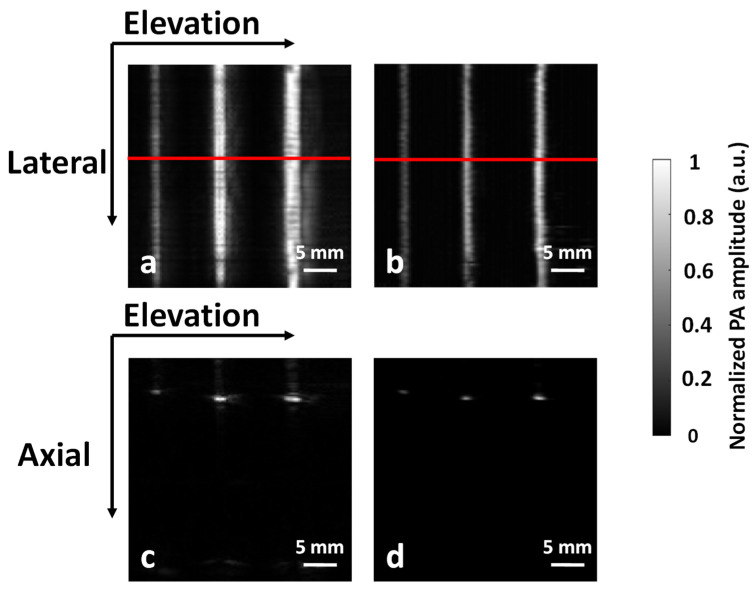
Imaging results of pencil leads of different diameters. (**a**) Input reconstructed by the 3D focal-line algorithm. (**b**) Combined (2D Deep-E and 3D Deep-E) output. (**c**) Cross-sectional image obtained from the location indicated by the red line in (**a**). (**d**) Cross-sectional image obtained from the location indicated by the red line in (**d**).

To further validate whether our algorithm can recover the true object size in an experimental environment, we imaged two phantoms to evaluate the performance. The first phantom was inserted with 0.5 mm pencil leads at 8 mm depth increments. The pencil leads were located 30 to 60 mm away from the transducer surface, mimicking the imaging distance of an in vivo experiment. Figure 5a,b show the depth-encoded MAP images for the input and output combined with 2D and 3D Deep-E, respectively. Here, the depth indicates the distance to the surface of the phantom. Figure 5c,d denote the cross-sections indicated by the red line in Figure 5a,b, respectively. Compared to the input (Figure 5a,c), the object in the output (Figure 5b,d) exhibited a much finer structure in both axial and elevation directions. It can also be seen that our approach performs well on objects at different depths, which is essential for deep tissue imaging. Table 3 lists the average diameter of all pencil leads quantified based on the full width at half maximum (FWHM). The error bar was quantified based on the diameters at different locations. The diameters obtained from the output image are very close to the ground truth in both directions. For the second phantom, three pencil leads with different diameters of 0.5, 0.9, and 2 mm were inserted at the same depth, which is 40 mm away from the transducer. Figure 6a,b show the MAP images for the input and output combined with 2D and 3D Deep-E, respectively. The red line in Figure 6a,b indicates the location of cross-sectional images shown in Figure 6c,d. Table 4 lists the quantified diameters of pencil leads along the elevation direction. While there are some variations, overall, the recovered diameters are closer to the ground truths (in comparison to the input). As will be mentioned later in the discussion, the accuracy can be further improved by training a larger dataset with a broader range of diameters. In addition, we also observed higher contrast-to-noise ratio due to reduced background noise in the output images.

### 3.3. Validation with In Vivo Data

**Figure 7:**
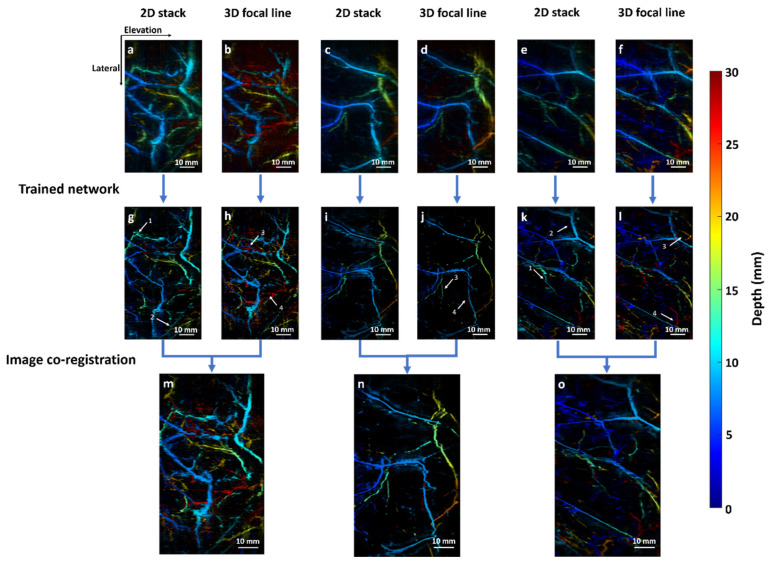
In vivo validation using human breast data. (**a**,**c**,**e**) 2D stack reconstructed images from three subjects, respectively. (**b**,**d**,**f**) 3D focal-line reconstructed images from the three subjects, respectively. (**g**,**i**,**k**) Output images from 2D Deep-E. (**h**,**j**,**l**) Output images from 3D Deep-E. (**m**,**n**,**o**) Final output after image co-registration. The color bar represents depth ranges from 0 to 30 mm.

Subsequently, we evaluated the performance of our approach using in vivo data obtained from human breast imaging experiments. Images from three subjects were selected. Similar to the simulation data, all the images are depth-encoded from blue to red. Figure 7a,c,e denote the 2D-stack reconstructed images from three subjects, respectively. In all cases, vessels from deep regions cannot be clearly revealed. Figure 7b,d,f denote the images reconstructed using the 3D focal-line algorithm. As expected, more vessels can be visualized from deep regions (colored in red). However, some vessels near the skin surface were not clearly visible. Figure 7g,i,k denote the outputs from the 2D Deep-E, and Figure 7h,j,l represent the outputs from 3D Deep-E. The elevation resolution has been significantly improved in all images. However, similar to data from the input, some vessels (arrows 1 and 2) are more visible in the 2D stack reconstructed result, while some (arrows 3 and 4) are more visible in 3D reconstructed images. The final co-registered images are shown in Figure 7m,n,o. It can be seen that these three images maintain the high elevation resolution from 2D/3D Deep E processing, and vessels extracted from both networks are clearly visible.

## 4. Discussion

Following the success of 2D Deep-E, we further developed 3D Deep-E and combined outputs from both 2D Deep-E and 3D Deep-E images to get the best from both algorithms. In 2D Deep-E, features reconstructed from 2D stack reconstruction are limited as it does not consider the receiving angle along the elevation direction. This inherent limitation leads to poor visualization of vessels extended along the lateral direction as well as features from deep regions. To address this issue, we combined reconstruction from both 2D stack and 3D focal-line reconstruction algorithms. In theory, applying 3D focal-line reconstruction alone would be sufficient to recover all the features [21]. However, 3D reconstruction is sensitive to the motion artifact induced by body movement and breathing. As a result, some vessels cannot be correctly reconstructed due to motion. By comparison, a 2D stack only reconstructs one frame at a time, mitigating the influence of motion. While 2D stack reconstruction is less sensitive to motion artifacts, it only reconstructs data in the axial and lateral plane, degrading image quality in deeper regions. Our approach effectively combines the benefits of both 2D and 3D reconstruction. Experimental results indicate that our method improves elevation resolution, recovers deeper vessels, and maintains the detailed vasculature from both reconstruction results. In addition, as we accounted for both Gaussian noise and EMI noise in training, the signal-to-noise ratio of the output image was significantly enhanced. 

**Figure 8:**
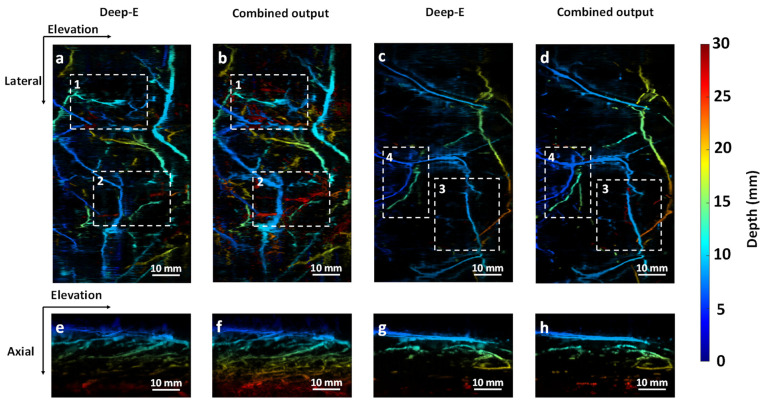
Comparison between 2D Deep-E and combined (2D Deep-E and 3D Deep-E) output. (**a**) Subject 1 output image from 2D Deep-E. (**b**) Subject 1 output image from the combined approach. (**c**) Subject 2 output image from 2D Deep-E. (**d**) Subject 2 output image from the combined approach. (**e**) Cross-section MAP image of (**a**). (**f**) Cross-section MAP image of (**b**). (**g**) Cross-section MAP image of (**c**). (**h**) Cross-section MAP image of (**d**).

A detailed comparison between the two approaches can be seen in Figure 8. Compared to Figure 8a,c, Figure 8b,d possess more vessels from deep regions, such as regions marked by dashed boxes 1, 2, and 3 (more red-colored vessels). In the meanwhile, vessels extended along the lateral direction are displayed in a more continuous manner (such as the region marked by dashed box 4). Figure 8e,f show the cross-sectional MAP images of Figure 8a,b, respectively. It can be seen that Figure 8f clearly contains more vessels in the deep region. Similar outcomes can be seen in Figure 8g,h. 

Despite the encouraging results, future studies are still needed to optimize our method. First, we did not quantify whether the quantitative information is preserved, especially after image co-registration. This issue could be addressed by studying how the co-registration process changes the image intensity and whether proper weighting can be applied to preserve the original signal distribution. Second, 3D focal-line reconstruction is more sensitive to motion artifacts, which cannot be avoided in the current setup. We can resolve this issue by using a higher pulse repetition frequency laser to shorten the scanning time and minimize the motion artifact. Third, vessels with large diameters in the input image might not be accurately recovered to the true size. This is due to the limited training dataset, which does not contain sufficient samples of large-diameter vessels. In the future, vessel samples with a broader diameter range could be utilized to address this issue. Finally, the vessel continuity can be further improved. As our approach optimized the image quality based on the cross-sectional image from the reconstructed matrix, connections with adjacent frames were not considered. The extracted features from each cross-section might have slight misalignment, degrading the continuity in the final 3D image. In the future, we can develop an algorithm to consider the connection between frames to improve continuity. 

It should be noted that, similar to other software-based approaches, our algorithm cannot achieve real super-resolution imaging as the hardware remains the same as in the conventional imaging [26]. However, compared to other approaches, such as deconvolution, our method has the following advantages. First, the processing speed is fast. After training, it takes less than 10 s to process reconstructed data. Second, it can recover the true diameter of the object, as shown in Figure 5b,d. In comparison, deconvolution based on a single point spread (PSF) function cannot recover the true size of objects at different depths as the PSF would change. Third, the system noise is built into the training. Therefore, our method is less affected by noise than deconvolution.

## 5. Conclusions

In this study, we developed a combined 2D Deep-E and 3D Deep-E approach to improve linear-array-based PACT. For 3D Deep-E training, we applied the virtual-point detector concept to generate the input data, mimicking images reconstructed from the 3D focal-line reconstruction algorithm. After obtaining output from 2D Deep-E and 3D Deep-E, we co-registered the two matrices to remove mismatches caused by the two reconstruction algorithms. The numerical validation demonstrated the method’s capability in elevation resolution enhancement, true feature size recovery, and noise reduction. In vivo validation further proved that our method can better recover deep vascular features and maintain detailed vasculature from both reconstruction algorithms. In summary, we further enhanced the performance of the Deep-E network by combining it with 3D focal-line reconstruction. Our results demonstrated improved image resolution and deeper vessel recovery. 

## Figures and Tables

**Table 1:** PSNR and SSIM of input and output in 9dB, 12dB, and EMI only.

Gaussian Noise Level	Input PSNR (dB)	Output PSNR (dB)	Input Cross-Section PSNR (dB)	Output Cross-Section PSNR (dB)	Input SSIM	Output SSIM	Input Cross-Section SSIM	Output Cross-Section SSIM
9 dB	5.54	17.61	10.78	29.93	0.014	0.756	0.001	0.994
12 dB	6.74	17.96	12.16	29.31	0.023	0.814	0.001	0.994
EMI only	11.85	20.13	19.49	38.78	0.051	0.859	0.009	0.995

**Table 2:** Diameter quantification along axial and elevation directions of simulated vasculature.

	Object 1 Diameter (mm)			Object 2 Diameter (mm)		
	Axial	Elevation	Average	Axial	Elevation	Average
Input	0.63	0.98	0.81	0.76	1.53	1.15
Output	0.26	0.27	0.27	0.32	0.43	0.38
Ground truth	0.27	0.29	0.28	0.32	0.51	0.41

**Table 3:** Diameter quantification along axial and elevation directions, and the contrast-to-noise ratio of pencil leads.

	Averaged Pencil Lead Diameter (mm)		Contrast-to-Noise Ratio
	Axial	Elevation	
Input	0.85 ± 0.08	1.82 ± 0.13	7.32
Output	0.56 ± 0.07	0.54 ± 0.04	12.53
Ground truth	0.50	0.50	

**Table 4:** Pencil lead diameter quantification along the transducer’s elevation direction.

Pencil Lead Diameter along the Trasnducer’s Elevation Direction (mm)			
Input	0.91 ± 0.09	1.92 ± 0.19	2.81 ± 0.42
Output	0.71 ± 0.07	0.93 ± 0.16	1.55 ± 0.11
Ground truth	0.50	0.90	2.00

## Data Availability

Publicly available scripts and datasets are uploaded to Github and the link is attached. https://github.com/wzheng26/3D-Deep-E.git. Accessed on: 16 September 2022. Data available upon request from authors.
